# Lower phase angle as a marker for poor prognosis in patients with chronic kidney disease: a cohort study

**DOI:** 10.3389/fnut.2025.1580037

**Published:** 2025-06-06

**Authors:** Yi-qin Chen, Hui-fen Chen, Yan Han, Yu-han Shen, Yi-dan Zhang, Li-zhe Fu, Fang Tang, Xu-sheng Liu, Yi-fan Wu

**Affiliations:** ^1^The Second Clinical College, Guangzhou University of Chinese Medicine, Guangzhou, China; ^2^Chronic Disease Management Outpatient Clinic, The Second Affiliated Hospital of Guangzhou, University of Chinese Medicine (Guangdong Provincial Hospital of Chinese Medicine), Guangzhou, China; ^3^Renal Division, The Second Affiliated Hospital of Guangzhou University of Chinese Medicine (Guangdong Provincial Hospital of Chinese Medicine), Guangzhou, China; ^4^Chinese Medicine Guangdong Laboratory, Hengqin, China

**Keywords:** chronic kidney disease, phase angle, bioimpedance, nutrition, cohort study

## Abstract

**Objective:**

Phase angle (PhA) obtained through bioimpedance analysis has been linked to mortality and malnutrition in dialysis patients. However, it remains unclear whether PhA is associated with renal prognosis in non-dialysis CKD patients.

**Methods:**

Two thousand two hundred two CKD patients were enrolled in the SMP-CKD cohort, Guangdong Provincial Hospital of Traditional Chinese Medicine from July 1, 2015 to May 31, 2024. Participants undertook bioimpedance measures, and the correlation between PhA and renal endpoint was analyzed. Analytical approaches include Cox proportional hazards analysis and group-based trajectory modeling. Composite outcome is defined as the first occurrence of >30% decline or <5 mL/min/1.73m^2^in eGFR, doubled of SCr from the baseline, initiation of continuous dialysis therapy or receipt of a kidney transplant, or all-cause mortality.

**Results:**

During a mean follow-up of time 2.5 years, 570(25.9%) participants reached the composite endpoint. In the multivariable Cox regression model, subjects belonging to higher quartiles of phase angle presented with a decreased risk of poor prognosis, showing 29 and 38% risk reductions in Q3 (aHR 0.71, 95%CI 0.55–0.93) and Q4 (aHR 0.62, 0.45–0.85) versus Q1 (both *p* < 0.05). When modeled in 2 groups according to the turning point of 5.0°, the adjusted hazard ratios (aHRs; 95% confidence intervals [CIs]) for broad-PhA group was 0.77(0.63, 0.95) compared with narrow-PhA group. The group-based trajectory modelling (GBTM) identified 4 trajectories, and the beneficial association remained consistent, with aHR (95% CIs) for group 2, group 3, group 4 were 0.69 (0.50–0.95), 0.59 (0.39–0.90), 0.47 (0.24–0.93), respectively, compared with group 1.

**Conclusion:**

Phase angle could be useful in determining nutritional status of CKD patients, lower phase angle is an independent risk factor for poor prognosis in CKD patients.

## Introduction

Chronic kidney disease (CKD) is a global health problem with an increasing prevalence globally, characterized by declined glomerular filtration rate of lower than 60 mL/min/1.73m^2^ or biomarkers signifying kidney injury. CKD is an irreversible and progressive disease, once the patients enter to end-stage renal disease, they had to rely on renal replacement therapy to prolong life, causing economic burden and reducing life-quality. Meanwhile, impaired renal function also causes uremia accumulation and metabolic abnormalities, which also increases the risk of cardiovascular disease and mortality (1).

Bioelectrical Impedance Analysis (BIA) is a generally considered low-cost, non-invasive technique used to measure body composition by applying weak alternating electrical currents through the body. This method can provide relatively comprehensive information about body composition parameters, such as lean body mass, fat mass, and total body water content. By analyzing these measurements in relation to established reference ranges, healthcare professionals may gain insights that could help evaluate a patient’s nutritional status. While bioimpedance-derived fluid status indices have long been associated with chronic kidney disease (CKD) progression, accumulating evidence has highlighted that muscle loss (sarcopenia) and musculoskeletal wasting are strongly correlated with adverse clinical outcomes in CKD patients (2). Nevertheless, traditionally used methods such as upper arm circumference measurements, skin fold measurements and body mass index (BMI) count provided limited information and lack uniformity, accuracy and integrity in evaluating muscle mass (3, 4).

Phase angle (PhA) has gained increasing attention as nutritional indicator for CKD patients (5, 6) and can be regarded as a signal of cellular health and fluid balance (7, 8). It is a raw measurement expressed in degrees (°) and is calculated using the arctangent function of the ratio between reactance (Xc) and resistance (R) (9). Xc corresponds to the capacitance of cell membrane which can block the passage or delay the time of alternating current, which means a more intact cell membrane signifies a higher capacitance; while the resistance value R is determined by intracellular and extracellular electrical resistance (5) and is closely related to the total water content in human body (Supplementary Figure S1), which means cells with a higher water content such as muscle cells correlate with lower resistance. For example, malnutrition causes an elevation in the ratio of extracellular water/intracellular water and extracellular water/cell mass (10, 11), which subsequently results in a decline in PhA due to decreased reactance. Thus, phase angle is a major role in determining cell integrity and is a predictor of body muscle mass, and has also been suggested to be the prognostic and nutritional indicator of several diseases (12–14). Previous studies have primarily concentrated on dialysis CKD patients (15, 16). In addition, research involving non-dialysis CKD patients have demonstrated the associations between PhA and sarcopenia, nutritional status, vascular calcification (17, 18); nevertheless, these studies are yet to uncover the definite association of PhA and the renal outcome of CKD patients. At present, the evidence supporting the correlation between phase angle PhA and CKD outcomes is limited in non-dialysis CKD populations. Therefore, the main aim of our research is to investigate the clinical significance of PhA in predicting renal outcomes among non-dialysis patients.

## Methods

### Patients and data collection

**Figure 1:**
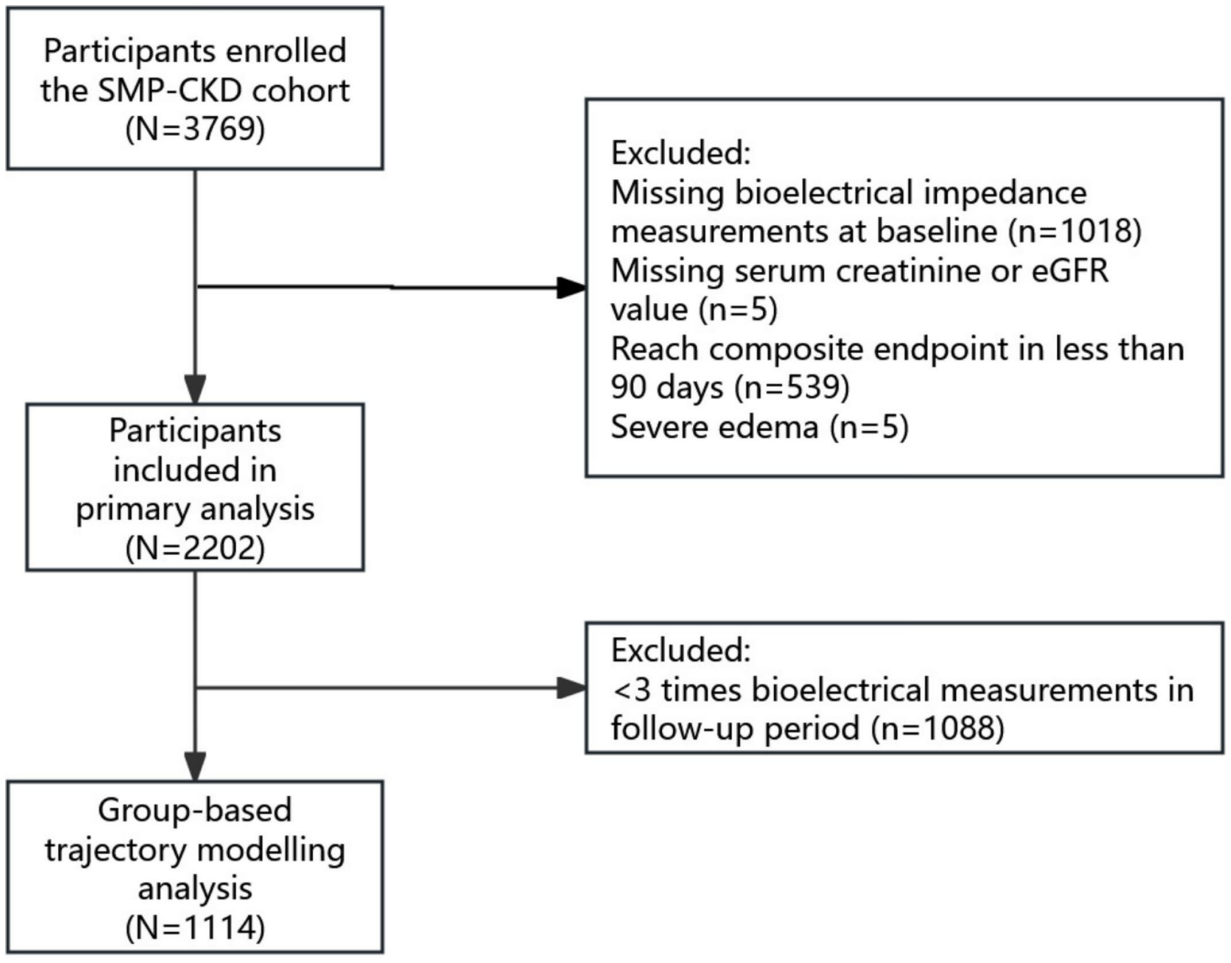
Study flowchart.

The Self-Management Program for Patients with Chronic Kidney Disease Cohort (SMP-CKD) (19) cohort study is an ongoing, multi-center and ambispective cohort study aimed at discovering the effects of patients’ self-management ability on CKD prognosis. For the present study, we retrospectively utilized data from CKD stages 1–5 patients enrolled in the SMP-CKD cohort between July 1, 2015, and May 31, 2024. All participants were recruited from the Guangdong Provincial Hospital of Traditional Chinese Medicine. CKD was diagnosed according to the KDIGO 2012 Clinical Practice Guidelines for the Evaluation and Management of Chronic Kidney Disease, which define CKD as the presence of kidney damage (e.g., albuminuria, abnormal urine sediment, or structural abnormalities on imaging) or a decreased glomerular filtration rate (GFR) of less than 60 mL/min/1.73 m^2^ for more than 3 months. Participants registered to the cohort underwent bioelectrical impedance analysis, laboratory evaluation and a questionnaire survey. The inclusion criteria included participants above 18 years old and diagnosed with CKD according to the KDIGO guidelines. Participants with incomplete BIA test or missing eGFR levels at baseline, those under 18 years old, or those with a survival time of less than 3 months were excluded (Figure 1). All participants signed the informed consent, and the study was approved by the Ethics Committee of Guangdong Provincial Hospital of Chinese Medicine (Ethics approval No. 2019–153-01; Chinese Clinical Trial Registry No. ChiCTR1900024633).

### Bioelectrical impedance analysis of body composition

Participants underwent body composition assessment using InBody770 (InBody Co., Ltd.) through direct segmental multi-frequency bioelectrical impedance analysis (DSM-BIA) at baseline and follow-up, with morning examinations conducted under standardized conditions: ≥2-h fasting, bladder/bowel evacuation, 10-min seated rest, and ambient temperature control (20–25°C). Contraindications excluded individuals with implanted electronic medical devices (e.g., pacemakers), while measurements were deferred post-exercise, or during the first 3 days of menstruation. Repeat assessments maintained identical posture and electrode placement to ensure measurement consistency. Height and weight values were measured and then manually entered into the InBody 770 device which automatically calculated BMI. The device further provided estimates of various body composition parameters, including phase angle, intracellular water (ICW), skeletal muscle mass (SMM), percent body fat (PBF), etc.

### Covariates

Data of covariates was collected through detailed questionnaires, medical records, and laboratory tests. Demographics collected by questionnaires included age(continuous) and gender (male or female). Comorbidities including hypertension, diabetes and hyperlipidemia, hyperuricaemia were defined as follows. Hypertension was defined as systolic blood pressure ≥140 mmHg or diastolic blood pressure ≥90 mmHg, or current use of antihypertensive medication. Diabetes was defined as fasting plasma glucose ≥7.0 mmol/L, HbA1c ≥ 6.5%, or current use of antidiabetic medication. Hyperlipidemia was defined as total cholesterol ≥6.2 mmol/L, LDL cholesterol ≥4.1 mmol/L, or current use of lipid-lowering medication. Hyperuricemia was defined as serum uric acid levels ≥420 μmol/L for men and ≥360 μmol/L for women. History of cardiovascular diseases was identified through cardiac ultrasound and electrocardiogram. Blood samples were collected in the morning within 2 weeks before and after the baseline date (enrollment date of the study), and routine laboratory tests were conducted to measure neutrophil, monocyte, lymphocyte, uric acid (UA), serum albumin (Alb), serum high-density lipoprotein (HDL), serum low-density lipoprotein (LDL), serum creatinine (Cr), urine albumin to creatinine ratio (UACR) and estimated glomerular filtration rate (eGFR). Prognostic nutritional index (PNI) and systemic inflammation response Index (SIRI) were also calculated (Supplementary Item S1).

### Composite outcome and follow-up

Composite outcome is defined as the first occurrence of >30% decline or <5 mL/min/1.73m^2^in eGFR, doubled of SCr from the baseline, initiation of continuous dialysis therapy or receipt of a kidney transplant, or all-cause mortality. CKD stages are calculated according to the CKD Epidemiology Collaboration (CKD-EPI) creatinine equation (20).

At semi-annual in-person visits, participants were queried about self-management knowledge, they also take laboratory tests and underwent BIA examinations.

### Statistical analysis

To account for missing data in the covariates, we employed multiple imputations and repeated analyses using the imputed dataset. For each CKD outcome, multiple imputations by chained equation were used under a ‘missing at random’ assumption, creating five imputed datasets (21). Predictive mean matching was used for continuous variables, and polytomous regression was used for categorical variables (21). The HR was estimated using Rubin’s formula (22). Correlations between body composition parameters were assessed using Pearson’s correlation coefficient (Supplementary Figure S2), while variance inflation factor (VIF) was used to evaluate the correlation between other parameters (Supplementary Table S2), then variables with high collinearity were excluded from covariates.

Baseline characteristics of the study population are described according to quartiles of phase angle. Continuous variables are expressed as mean ± standard deviation (SD) for normal distributions and median (interquartile ranges) for non-normal distributions, while categorical variables are expressed as percentages (%). The profile distribution between different quartile groups was analyzed using the Chi-square test. Statistical comparisons of continuous variables were performed using the Kruskal-Wallis test for non-normally distributed data and one-way ANOVA for normally distributed data.

To identify the turning point value and nonlinearity of the association between PhA and CKD outcome, we also applied restricted cubic spline (RCS) regression with 3 knots (at 10th, 50th, and 90th centiles) and adjust for relevant covariates. We generated Kaplan–Meier curves and Cox regression model to test the association of phase angle with clinical outcomes. Proportional hazards test based on weighted residuals was applied to check whether the proportional hazard assumptions of Cox regression were violated. Since the result of Schoenfeld (with the survminer R package) showed violation of the assumption in some observed variables, we transformed the variables into time-dependent covariates conducted Cox model to calculate the hazards ratio (HRs). Adjustment for relevant covariates including age, sex, BMI, comorbidities and laboratory tests were performed in adjusted models. Subgroup analyses was conducted to examine the presence of significant interactions of these covariates with the association between PhA and CKD outcomes. Multivariable-adjusted Cox models with strata of sex, age, CKD stages, BMI, SMM, with or without certain comorbidities were constructed. Forest plots were used to show the hazards ratios (HRs) with 95% (CIs) and interaction with subgroups.

To investigate the association of CKD outcome with trajectory of phase angle, we implement Group-based trajectory modeling (GBTM), in which we identify distinct trajectories of PhA as a function of follow-up period at each visit. Given that trajectory analysis is more stable for participants with 3 or more observations over time, we excluded participants with less than 3 times accessible data of BIA measurements. Initially, we fitted models ranging from a single trajectory group to five trajectory groups by using polynomial models (up to cubic) for each cognitive outcome. Subsequently, by comparing Bayesian Information Criteria (BIC) and Akaike’s Information Criterion (AIC) values, we select the best-fitted model (Supplementary Table S5). Additionally, a robust model fit was suggested by an average posterior probability of assigning participants to a specific group exceeding 70%. Consequently, models that exhibited a membership of more than 5% in each trajectory group were chosen for further consideration.

All statistical tests were two-sided, and *p* values <0.05 were considered statistically significant. GBTM analyses was conducted by SAS (V9.4), and the rest were performed using R version 4.4.1(R Foundation).

## Results

### Baseline characteristics of the study population

We finally included 2,202 patients (mean age 52 years old; 55.1% men) from CKD 1–5, with a median phase angle of 5.0°. The PhA values of the quartiles were 4.1° (3.8–4.3), 4.7° (4.6–4.8), 5.2° (5.1–5.4), and 5.9° (5.7–6.2), respectively. As shown in Table 1, participants in the highest quartile of PhA groups were more likely to be younger, man; less likely to have hypertension, diabetes, hyperuricemia and cardiovascular disease history; have higher levels of eGFR, uric acid, hemoglobin (Hb) and BMI, and lower levels of low-density lipoprotein cholesterol (LDL-C), high-density lipoprotein cholesterol (HDL-C), systolic blood pressure (SBP) and mean arterial pressure (MAP) and earlier stages of CKD (all *p* < 0.05).

**Table 1:** Baseline characteristic grouped by quartiles of phase angle.

Variables	Total	PhA<4.5°(Q1)	4.5° ≤ PhA<5°(Q2)	5° ≤ PhA< 5.6°(Q3)	PhA≥5.6°(Q4)	*p* value
	*N* = 2,202	*N* = 533	*N* = 496	*N* = 583	*N* = 503	
Man, *n* (%)	1,213 (55.1%)	216 (40.5%)	195 (39.3%)	318 (54.5%)	484 (82.0%)	<0.001
Age, years	52.0 (38.1–64.1)	62.7 (49.5–72.4)	56.3 (42.1–66.2)	51.0 (39.2–61.1)	42.2 (33.5–51.8)	<0.001
CKD stage, *n* (%)						<0.001
1	469 (21.3%)	102 (19.1%)	95 (19.2%)	141 (24.2%)	131 (22.2%)	
2	551 (25.0%)	79 (14.8%)	106 (21.4%)	143 (24.5%)	223 (37.8%)	
3	679 (30.8%)	185 (34.7%)	154 (31.0%)	185 (31.7%)	155 (26.3%)	
4	299 (13.6%)	94 (17.6%)	87 (17.5%)	72 (12.3%)	46 (7.80%)	
5	204 (9.26%)	73 (13.7%)	54 (10.9%)	42 (7.20%)	35 (5.93%)	
Hypertension, *n* (%)	1,196 (54.3%)	349 (65.5%)	278 (56.0%)	300 (51.5%)	269 (45.6%)	<0.001
Diabetes, *n* (%)	469 (21.3%)	199 (37.3%)	125 (25.2%)	88 (15.1%)	57 (9.66%)	<0.001
Hyperlipidemia, *n* (%)	729 (33.1%)	197 (37.0%)	158 (31.9%)	172 (29.5%)	202 (34.2%)	0.053
Hyperuricemia, *n* (%)	1,117 (50.7%)	250 (46.9%)	233 (47.0%)	283 (48.5%)	351 (59.5%)	<0.001
Cardiovascular disease, *n* (%)	184 (8.36%)	71 (13.3%)	44 (8.87%)	41 (7.03%)	28 (4.75%)	<0.001
SBP, mmHg	127 (120–136)	130 (122–141)	128 (120–137)	126 (118–134)	125 (118–132)	<0.001
DBP, mmHg	75.0 (69.0–80.0)	74.5 (68.0–80.0)	75.0 (70.0–80.0)	76.0 (69.0–80.0)	76.0 (70.0–80.9)	0.144
MAP, mmHg	92.7 (86.7–98.3)	93.3 (87.7–99.7)	92.7 (86.7–98.1)	92.3 (85.9–97.7)	92.3 (86.5–97.8)	0.037
Cr, μmol/L	114 (83.0–176)	124 (83.0–225)	115 (80.0–194)	110 (78.0–164)	112 (91.0–144)	0.008
eGFR, ml/min/1.73 m^2^	55.4 (31.6–84.2)	44.1 (22.1–73.6)	51.5 (25.0–83.2)	58.7 (36.2–89.3)	66.8 (46.6–86.0)	<0.001
HDL-C, mmol/L	1.32 (1.14–1.51)	1.36 (1.16–1.59)	1.33 (1.18–1.51)	1.32 (1.14–1.51)	1.30 (1.10–1.45)	<0.001
LDL-C, mmol/L	3.30 (2.84–3.80)	3.42 (2.90–4.00)	3.27 (2.77–3.77)	3.29 (2.82–3.80)	3.27 (2.89–3.76)	0.034
UA, mmol/L	421 (357–481)	421 (348–473)	411 (347–477)	420 (350–476)	429 (367–498)	<0.001
Alb, g/L	43.3 (39.0–46.2)	39.2 (32.7–43.5)	43.0 (39.6–45.3)	43.7 (40.4–46.2)	45.3 (42.3–47.9)	<0.001
PNI	52.8 (48.0–57.0)	47.9 (42.2–53.3)	52.3 (49.0–56.5)	53.4 (49.6–56.3)	55.8 (52.2–59.4)	<0.001
SIRI	1.00 (0.65–1.55)	1.07 (0.68–1.70)	1.03 (0.64–1.53)	0.97 (0.66–1.44)	0.95 (0.64–1.46)	0.029
UACR, mg/g	612 (167–1776)	1,428 (397–3,732)	722 (207–1,621)	550 (171–1,387)	338 (98.7–939)	<0.001
BMI (kg/m^2^)	23.0 (20.7–25.4)	21.9 (19.8–24.2)	22.5 (20.3–25.0)	23.2 (21.0–25.6)	24.0 (22.0–26.5)	<0.001
TBW (L)	32.7 (28.0–37.8)	29.3 (26.0–34.8)	30.0 (26.8–35.0)	32.9 (28.5–37.6)	36.9 (33.2–40.0)	<0.001
ICW (L)	20.0 (17.1–23.2)	17.7 (15.6–20.8)	18.3 (16.3–21.2)	20.2 (17.6–23.0)	23.0 (20.7–24.9)	<0.001
ECW (L)	12.7 (10.9–14.6)	11.7 (10.3–14.0)	11.7 (10.4–13.7)	12.7 (10.9–14.5)	13.9 (12.5–15.1)	<0.001
BFM (kg)	16.4 (12.2–21.2)	15.3 (11.5–20.1)	16.8 (12.4–20.8)	16.8 (12.9–22.3)	16.6 (12.3–21.4)	0.005
SLM (kg)	41.9 (35.8–48.4)	37.5 (33.1–44.3)	38.4 (34.2–44.7)	42.1 (36.6–48.1)	47.5 (42.7–51.5)	<0.001
FFM (kg)	44.3 (38.0–51.2)	39.8 (35.3–47.0)	40.8 (36.4–47.3)	44.6 (38.8–51.0)	50.3 (45.2–54.5)	<0.001
SMM (kg)	24.1 (20.2–28.2)	21.0 (18.3–25.2)	21.9 (19.3–25.6)	24.3 (20.9–28.0)	28.0 (24.9–30.5)	<0.001
PBF (%)	27.1 ± 8.43	27.5 ± 9.23	28.4 ± 8.19	27.5 ± 8.41	25.1 ± 7.55	<0.001
ECW/TBW (%)	0.39 (0.38–0.39)	0.40 (0.39–0.41)	0.39 (0.39–0.39)	0.38 (0.38–0.39)	0.38 (0.38–0.38)	0.000
VFA (cm^2^)	72.3 (53.7–98.9)	72.0 (55.4–102)	76.2 (56.7–100)	72.3 (54.4–103)	68.5 (49.2–91.8)	<0.001
BCM (kg)	28.7 (24.4–33.2)	25.3 (22.3–29.8)	26.2 (23.4–30.3)	28.9 (25.2–33.0)	33.0 (29.6–35.7)	<0.001
PhA (°)	5.00 (4.50–5.60)	4.10 (3.70–4.30)	4.70 (4.60–4.80)	5.20 (5.10–5.40)	5.90 (5.70–6.20)	<0.001

The values for categorical variables are given as numbers (percentage); values for continuous variables are given as median [interquartile range] or mean ± standard deviation. Abbreviations: PhA, phase angle; CKD, chronic kidney disease; SBP, systolic blood pressure; DBP, diastolic blood pressure; MAP, mean arterial pressure; Cr, serum creatinine; eGFR, estimated glomerular filtration rate; LDL-C, low-density lipoprotein cholesterol; HDL-C, high-density lipoprotein cholesterol; UA, uric acid; SIRI, systemic inflammation response index; PNI, prognostic nutritional index; UACR, urine albumin/creatinine ratio; Alb, serum albumin; BMI, body mass index; ECW, Extracellular Water; ICW, Intracellular Water; BFM, Body Fat Mass; SLM, Soft Lean Mass; FFM, Fat-Free Mass; SMM, Skeletal Muscle Mass; PBF, Percent Body Fat; ECW/TBW, Extracellular Water/Total body water; VFA, Visceral Fat Area; BCM, Body Cell Mass.

### Association of PhA and CKD outcome

During the median follow-up period of 2.5 years, 570 (25.9%) patients reached the composite endpoint. Unadjusted Cox analysis revealed a significant inverse relationship between phase angle quartiles and composite endpoint risk, with hazard ratios decreasing progressively across ascending quartiles (Q2: 0.61; Q3: 0.52; Q4: 0.46) and per 1° increment in phase angle (HR 0.65, 95% CI 0.58–0.72; *p* < 0.001). In the fully adjusted model (Model 3), higher phase angle quartiles demonstrated clinically relevant protective effects, with adjusted hazard ratios of 0.71 (95% CI 0.55–0.93, *p* = 0.012) for Q3 and 0.62 (95% CI 0.45–0.85, *p* = 0.003) for Q4 compared to the reference quartile (Q1). The continuous analysis further confirmed this association, showing a 16% risk reduction per 1° increase in phase angle (aHR 0.84, 95% CI 0.72–0.98, *p* = 0.027).

**Figure 2:**
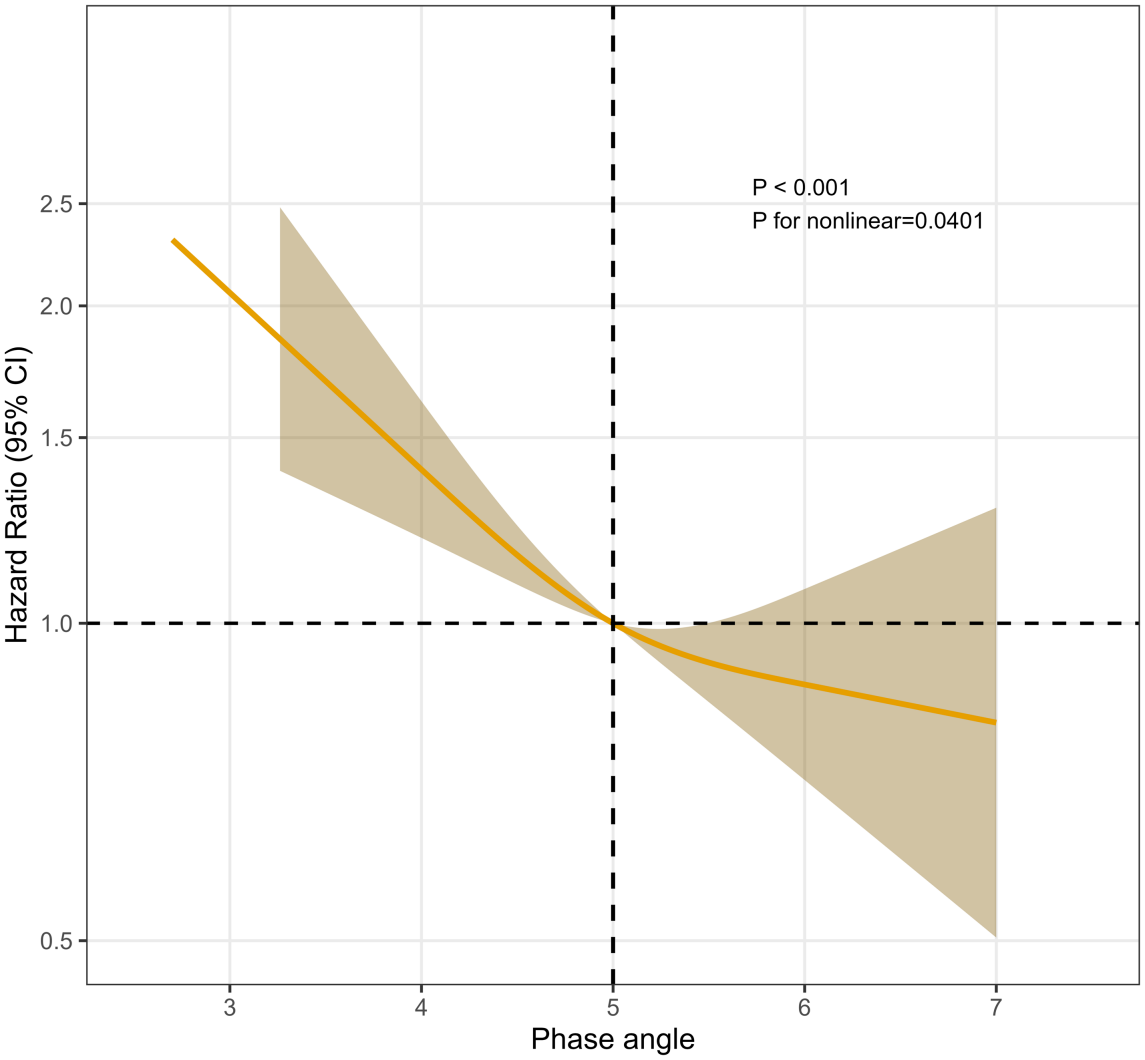
Association between phase angle and CKD outcome. CKD, chronic kidney disease.

Restricted cubic spline curve analysis displayed a non-linear and inverse relationship between PhA and CKD composite endpoint as shown in Figure 2. Based on the calculated turning point of 5.0°, we categorized the participants into two groups: narrow-PhA and broad-PhA, and subsequently analyzed their basic information along with BIA parameters (Supplementary Table S3).

**Figure 3:**
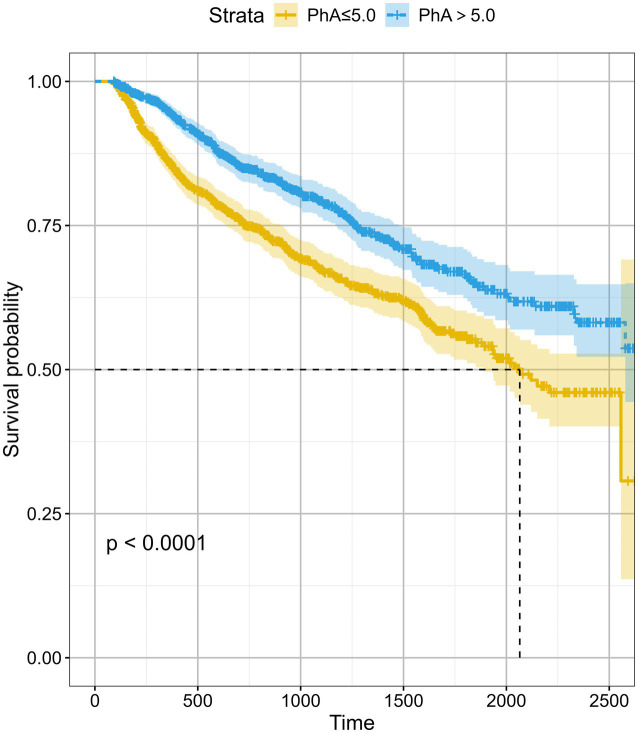
Kaplan–Meier survival curves for composite renal endpoint of the study participants stratified by the turning point of phase angle (PhA ≤ 5° vs. >5°). PhA, phase angle.

To investigate the relationship between CKD outcome and the narrow/broad PhA groups more thoroughly, we conducted a multivariate Cox regression analysis, adjusting for demographics and other covariates across three models (Table 2). In the final adjusted model, participants in the broad-PhA group had 23% lower risk for composite outcome (aHR: 0.77; 95% CI: 0.63–0.95). This association was also confirmed using the Kaplan–Meier curves (Figure 3).

**Table 2:** Multivariate Cox regression models showing the association between phase angle and CKD composite outcomes.

Phase Angle	Non-adjusted model		Model 1		Model 2		Model 3	*P* value
	HR (95%CI)	*P* value	HR (95%CI)	*P* value	HR (95%CI)	*P* value	HR (95%CI)	
Quartile								
Q1: PhA < 4.5° (Reference)								
Q2: 4.5° ≤ PhA < 5.0°	0.61(0.49,0.75)	<0.001	0.61(0.49,0.77)	<0.001	0.65(0.52,0.82)	<0.001	0.83(0.65, 1.05)	0.117
Q3: 5° ≤ PhA < 5.6°	0.52(0.42,0.65)	<0.001	0.49(0.39,0.62)	<0.001	0.53(0.41,0.67)	<0.001	0.71(0.55,0.93)	0.012
Q4: ≥ 5.6°	0.46(0.36,0.58)	<0.001	0.40(0.31,0.53)	<0.001	0.44(0.33,0.58)	<0.001	0.62(0.45,0.85)	0.003
Continuous								
Per 1° increase	0.65(0.58, 0.72)	<0.001	0.59(0.53,0.67)	<0.001	0.68(0.61, 0.76)	<0.001	0.84(0.72, 0.98)	0.027
Turning point								
Narrow (≤5°, Reference)								
Broad (>5°)	0.61(0.51,0.72)	<0.001	0.57(0.48,0.69)	<0.001	0.61(0.51,0.74)	<0.001	0.77(0.63, 0.95)	0.014

Model 1: adjusted for age and sex; Model 2: Model 1 plus body mass index, percent body fat, skeletal muscle mass, comorbidities (hypertension, diabetes, cardiovascular disease); Model 3: Model 2 plus laboratory measurements (urine albumin/creatinine ratio, high-density lipoprotein cholesterol, low-density lipoprotein cholesterol, uric acid, estimated glomerular filtration rate), prognostic nutritional index, systemic inflammation response index. PhA, phase angle; HR, hazard ratio; 95%CI, 95% confidence interval.

### The association of PhA trait trajectories and CKD composite outcomes

**Figure 4:**
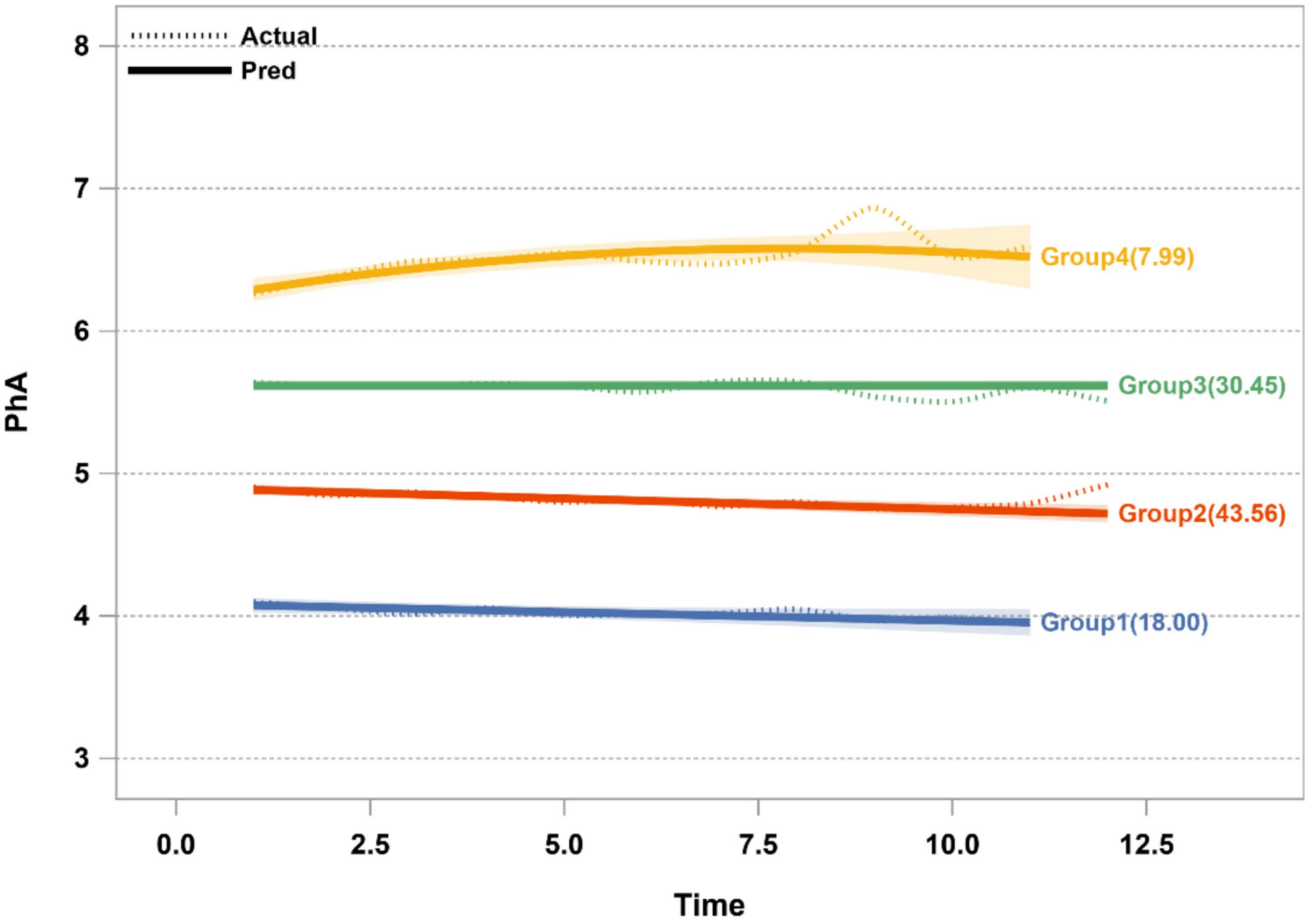
Mean trajectories of phase angle by increasing time among CKD patients. The vertical axis represents the level of PhA, the horizontal axis represents time (taking 6 months as one unit). 1,114 participants were included for GBTM analysis, with 196(18.00%), 491(43.56%), 341(30.45%) and 86(7.99%) participants divided into Group 1, Group 2, Group 3 and Group 4, respectively. PhA, phase angle.

The GBTM yielded four trajectory models as the best to fit to the data, plotted by months at each visit: class1, “persistently low” (n = 196, 17.5%); class2, “persistently moderately low” (*n* = 491, 44.1%); class 3, “persistently moderately high” (*n* = 341, 30.6%); and class 4, “persistently high” (*n* = 86, 7.7%) (Figure 4). The maximum likelihood estimates for the final four-group trajectory model are summarized in Supplementary Table S5. The baseline characteristics of participants in each trajectory group for phase angle are presented in Supplementary Table S4.

Table 3 summarizes the results from the multivariate Cox regression examining phase angle with CKD outcome. In the fully adjusted model (Model 3) incorporating clinical biomarkers and nutritional indices, participants in higher PhA trajectory groups demonstrated substantially lower composite outcome risks compared to the reference group, with fully multivariable-adjusted HR (95%CI) for group 2, group 3, group 4 of 0.69 (0.50–0.95), 0.59 (0.39–0.90),0.47 (0.24–0.93), respectively.

**Table 3:** Multivariate regression Cox models showing the association between phase angle trajectories and CKD composite outcomes.

Phase angle	Non-adjusted model		Model 1		Model 2		Model 3	*P* value
	HR (95%CI)	*P* value	HR (95%CI)	*P* value	HR (95%CI)	*P* value	HR (95%CI)	
Trajectories								
Group1 (Reference)								
Group2	0.67(0.51,0.90)	0.007	0.67(0.50,0.91)	0.009	0.70(0.52,0.95)	0.021	0.69(0.50,0.95)	0.026
Group3	0.59(0.45,0.83)	0.002	0.56(0.39,0.81)	0.001	0.54(0.37,0.78)	0.001	0.59(0.39,0.90)	0.014
Group4	0.38(0.22,0.68)	0.001	0.33(0.18,0.61)	<0.001	0.31(0.17,0.59)	<0.001	0.47(0.24,0.93)	0.029

Model 1: adjusted for age and sex; Model 2: Model 1 plus body mass index, percent body fat, skeletal muscle mass, comorbidities (hypertension, diabetes, cardiovascular disease); Model 3: Model 2 plus laboratory measurements (urine albumin/creatinine ratio, high-density lipoprotein cholesterol, low-density lipoprotein cholesterol, uric acid, estimated glomerular filtration rate), prognostic nutritional index, systemic inflammation response index. HR, hazard ratio; 95%CI, 95% confidence interval.

### Subgroup analysis

**Figure 5:**
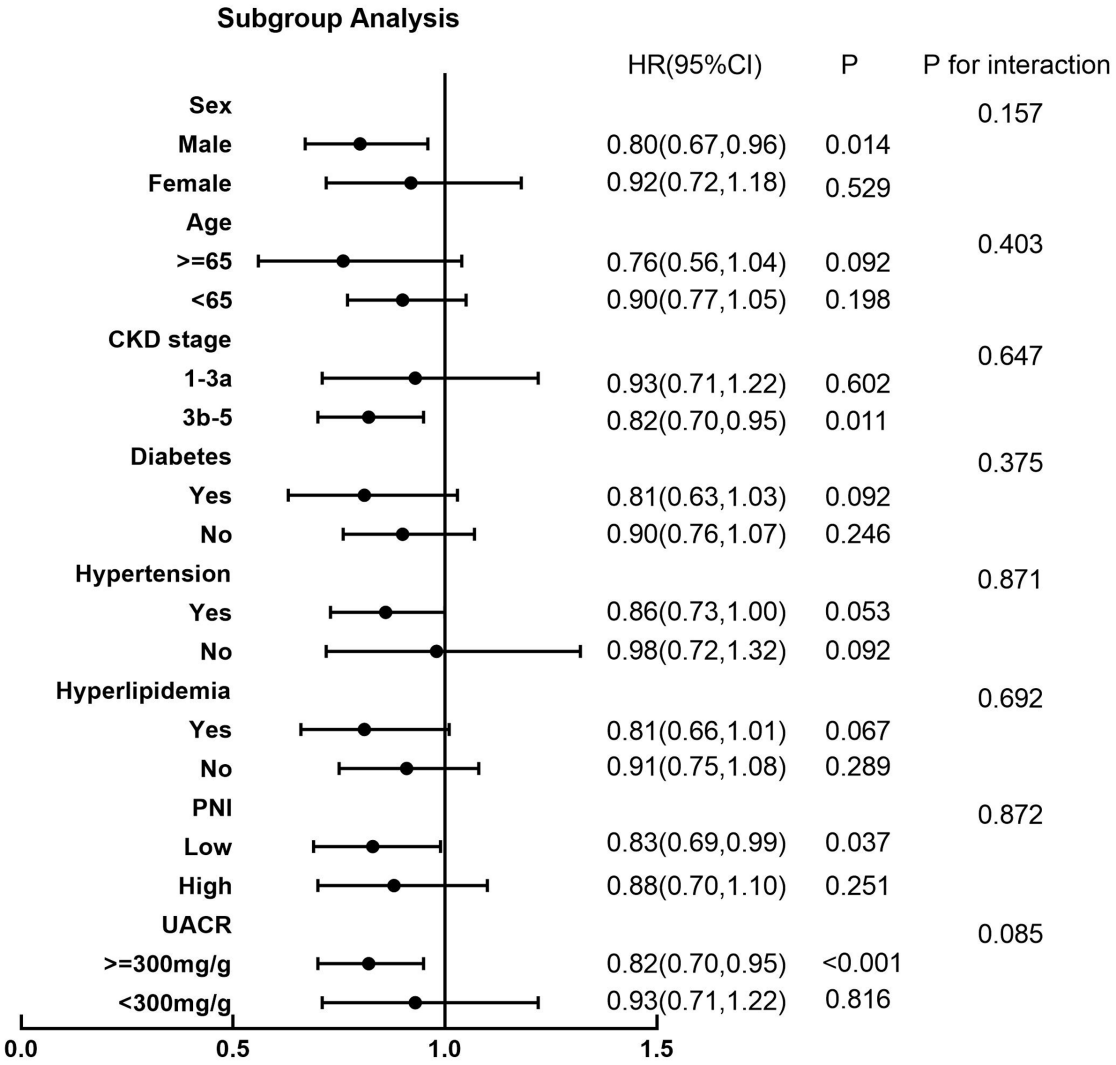
Forest plot of subgroup analysis The aHRs were adjusted for age, sex, body mass index, percent body fat, skeletal muscle mass, comorbidities (hypertension, diabetes, cardiovascular disease), laboratory measurements (urine albumin/creatinine ratio, high-density lipoprotein cholesterol, low-density lipoprotein cholesterol, uric acid, estimated glomerular filtration rate), prognostic nutritional index, systemic inflammation response index. CKD, chronic kidney disease; PNI, prognostic nutritional index; UACR, urine albumin/creatinine ratio; HR, hazard ratio; BMI, body mass index; HR, hazard ratio; 95%CI, 95% confidence interval.

A subgroup analysis was stratified by age, sex, CKD stage, UACR, hypertension, diabetes, hyperlipidemia, and PNI based on Model 3. As illustrated in Figure 5, the findings showed no interaction effect in the subgroup analysis (*p* > 0.05).

## Discussion

In our cohort study of non-dialysis CKD patients, we demonstrated that low phase angle as well as phase angle with ‘persistently low trajectories’ was significantly associated with higher risk of poor renal outcome. The finding indicate that phase angle could be a convenient, effective, and potential biomarker of risk in individuals with non-dialysis CKD. This association was independent of eGFR, hypertension or other risk factors for composite renal endpoint and was not modified by age or sex. In order to observe the longitudinal relationships, we employed GBTM models to fit PhA trajectories, and the findings revealed clear disparities in the baseline PhA levels, yet modest variations in the slope between the four trajectory groups. Besides, the GBTM model has good fitting parameters (Supplementary Table S5). The result indicated that individuals who possess a broad range of PhA tend to maintain at an elevated level throughout the disease course. This approach helped identify groups of individuals who experienced similar degrees of phase angle over time, whereas linear mixed models primarily concentrate on the average trends within the population. Moreover, it should also be noted that the result can also vary from different populations. Notably, we described the turning point is 5.0°, providing a specific and reliable criterion for predicting poor CKD outcome.

Various studies regarding the relationship between PhA and cardiovascular risk factors, nutrition, or sarcopenia have been conducted across different populations (18, 23, 24). As for dialysis patients or those who have undergone kidney transplantation, PhA can serve as a valuable nutritional indicator and has been linked to various health issues such as protein-energy wastage (PEW), malnutrition, and cardiovascular diseases (25–29). However, when it comes to the association between PhA and renal endpoints in non-dialysis CKD patients, there are especially fewer studies compared to those conducted on hemodialysis patients. Notably, our results showed a minor discrepancy with prior study outcomes. Chronic Renal Insufficiency Cohort (CRIC study) (23) revealed the association of lower phase angle and composite renal endpoint is not statistically significant in the multi-adjusted model. Similarly, a Spanish study (26) (307 advanced CKD participants) showed a positive association between phase angle and mortality risk in univariate analyses, but the effect was nullified after multivariable adjustment. However, the former study utilized single frequency Quantum II bioelectrical impedance, with lower accuracy than multi-frequency analyzer, while the latter study focused on later stages of CKD and featured a relatively limited sample size. The aforementioned factors, combined with variations in population characteristics, may explain why these studies failed to conclude that PhA is an independent indicator in the outcomes of CKD as our studies on the southern Chinese population.

PhA goes beyond merely quantifying body compartments, offering insights into hydration status, cellular mass, and the integrity of cell membranes (30). As such, PhA serves as an indicator of cellular health status (31) and can be affected by various pathologies. The pathophysiological mechanism of CKD likely involves uremic toxin accumulation in renal failure, which induces membrane lipid peroxidation and subsequent cellular dysfunction (32). In addition, inflammation, immune responses as well as metabolism issues also create free radicals or alter signal transduction, disrupting membrane structure further (33). From a nutritional perspective, CKD patients often develop a unique nutritional imbalance characterized by increasing requirement in energy and reduction in appetite due to catabolism and chronic inflammation, which ultimately resulted in reduced nutritional supply, causes deficiencies in fatty acids, vitamins, and minerals crucial for membrane maintenance and contribute to a decrease in phase angle (34, 35).

PhA serve as a new biomarker to diagnose malnutrition in an early stage in order to initiate nutritional interventions or provide appropriate treatment. Though it is influenced by various factors such as hydration status, inflammation, and the proportion of muscle and fat mass (30, 36, 37), however, it is particularly influenced by malnutrition. One Chinese study on non-dialysis CKD patients revealed that PhA is 6.03° and 4.88° in non-malnutrition and malnutrition patients, respectively (24). Besides, PhA is found to be associated with and protein-energy wastage (PEW), an important indicator in clinical and nutritional management of CKD and has been proved to be strongly relevant to mortality rate in the population (38, 39). Several studies have reported significant association of PhA and various diagnostic factors of PEW in hemodialysis patients (40–42). Furthermore, PhA is also an indicator of sarcopenia. The study in Brazil found that PhA is 4.5° for sarcopenic group and 5.6° for non-sarcopenic group (18). Additionally, studies revealed the correlation of PhA and traditional somatometry for evaluating muscle strengths. One study evaluated the relationship between PhA and the components of sarcopenia, and suggested that PhA can not only predict the presence of sarcopenia, but was also linearly related to handgrip strength (HGS), 6 m gait speed (GS) and skeletal muscle mass index (SMI) (43). Based on the above literature, if a narrower phase angle is observed in clinical practices, it can be valuable to assess the patient’s nutritional status concurrently. This can be achieved through multi-faceted approaches, including examining patient’s 3-day diet diary, presence of PEW, measuring grip strength and skinfold thickness, etc. Additionally, improvements in nutritional status should be sought through dietary and exercise adjustments or other relevant interventions. Clinicians may inquire them about gastrointestinal symptoms to identify whether they are adhering to incorrect protein restrictions and assist them in developing healthy eating habits. Besides, personalized exercise prescriptions are based on patients’ physical ability, for instance, adding resistance exercise and aerobic exercise can be helpful for patients with better physical ability, while frail patients are recommended to start with gentle training such as yoga and Baduanjin, combined with functional training. These measures aim to broader phase angle as well as improving patients’ quality of lives, and consequently enhance the prognosis of CKD.

There are some apparent strengths in our study. It is the first time to examine data from a relatively large cohort of non-dialysis CKD adults over an extended nine-year period in southern China, coupled with a thorough assessment of kidney function and body composition. As the first longitudinal study among Chinese non-dialysis CKD patients using trajectory analyses, we enabled the identification of distinct groups of individuals who exhibited similar phase angle levels and patterns over time. Our study contributes further knowledge of the correlation between changes in phase angle over time and CKD outcomes within the Chinese population.

The study also has several limitations. First, as a single-center Chinese cohort, its findings may lack generalizability to other populations, necessitating validation through large-scale multicenter studies. Secondly, incomplete data on grip strength, dietary intake, and C-reactive protein (CRP) levels limited direct assessment of nutritional-inflammation interactions. To address this, we employed surrogate inflammatory indices (SIRI/PNI) and propose incorporating standardized metrics like the Malnutrition-Inflammation Score (MIS) in future research to strengthen nutritional evaluation frameworks. Thirdly, the impact of primary kidney diseases on outcomes remains unexplored due to substantial missing data in protopathy documentation. Therefore, more researches are needed to fully comprehend the complex relationship between PhA and CKD progression.

## Conclusion

In conclusion, the cohort study showed that lower phase angle is associated with higher risk of CKD composite endpoint, with a turning point value of 5.0°. Phase angle, as an easily accessible indicator, may help assessing the nutritional condition and renal prognosis as well as guiding implementation of interventions in clinical practice.

## Data Availability

The datasets presented in this article are not readily available because due to the nature of this research, participants of this study did not agree for their data to be shared publicly, so supporting data is not available. Requests to access the datasets should be directed to wuyifan007@gzucm.edu.cn.
