# Supplementary material for: Lower phase angle as a marker for poor prognosis in patients with chronic kidney disease: a cohort study
Source: Front Nutr. 2025 Jun 6;12:1580037. doi: 10.3389/fnut.2025.1580037 (PMC12179792; doi:10.3389/fnut.2025.1580037)
Supplement: Supplementary file 1 [file Table_1.DOCX]

**Supplementary Materials**

Figure S1 A simplified model of schematic diagram of phase angle


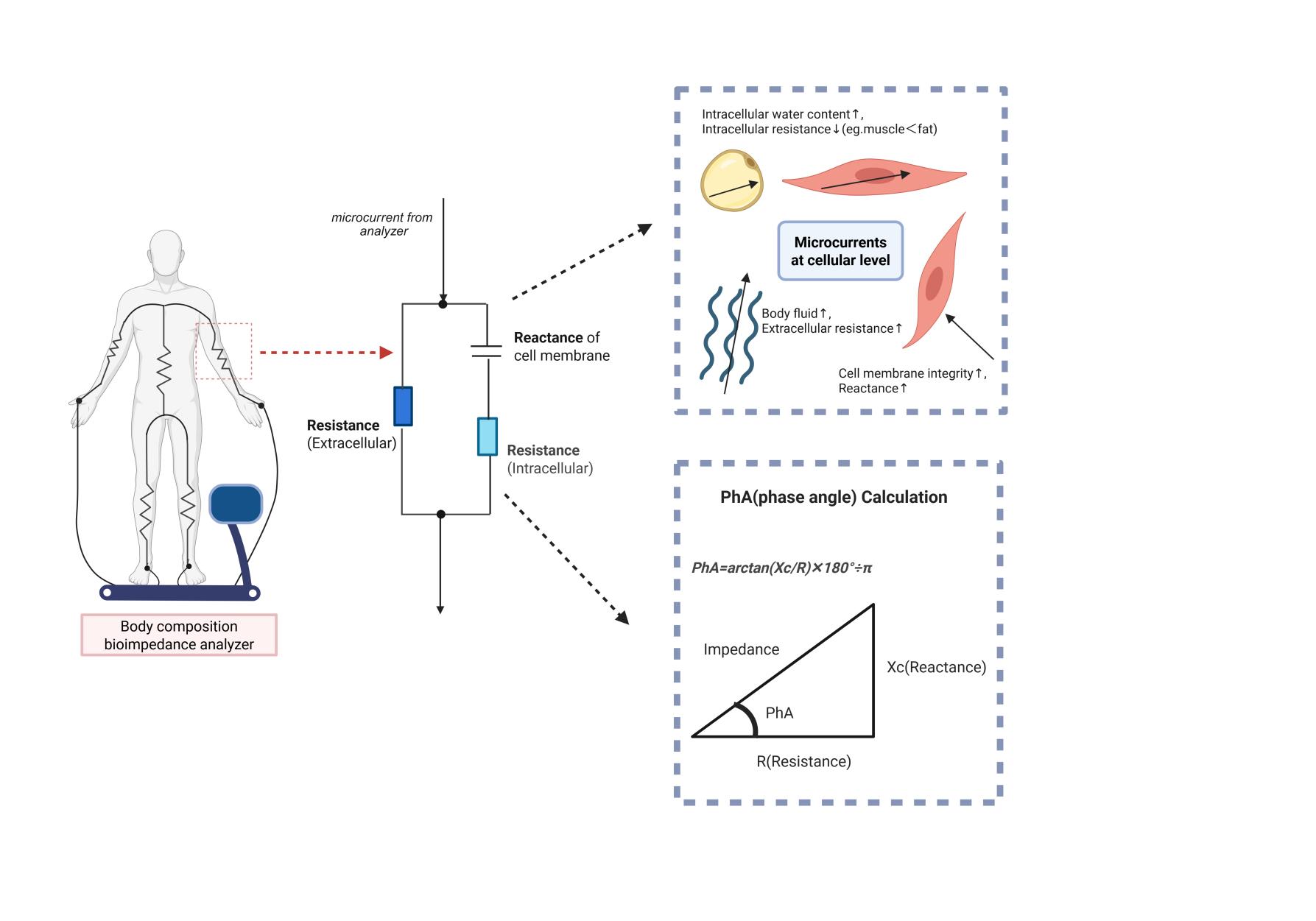


Figure legend: Diagram illustrating the measurement principles of bioimpedance analyser(BIA). BIA input alternating current electrical currents into the body through electrode slice, and form a circuit connecting resistance and reactance. Higher water content in the cell results in lower extracellular resistance, so the muscle cell has a significantly lower resistance than the fat cell. When body fluid increases, extracellular resistance also rise. Reactance depends on the integrity of cell membrane, the more complete the membrane, the higher the reactance value. Phase angle (PhA) is calculated by arctangent function of the ratio of reactance to resistance, times 180° times π.

Figure S2 Correlation matrix of body composition parameters (Pearson correlation)


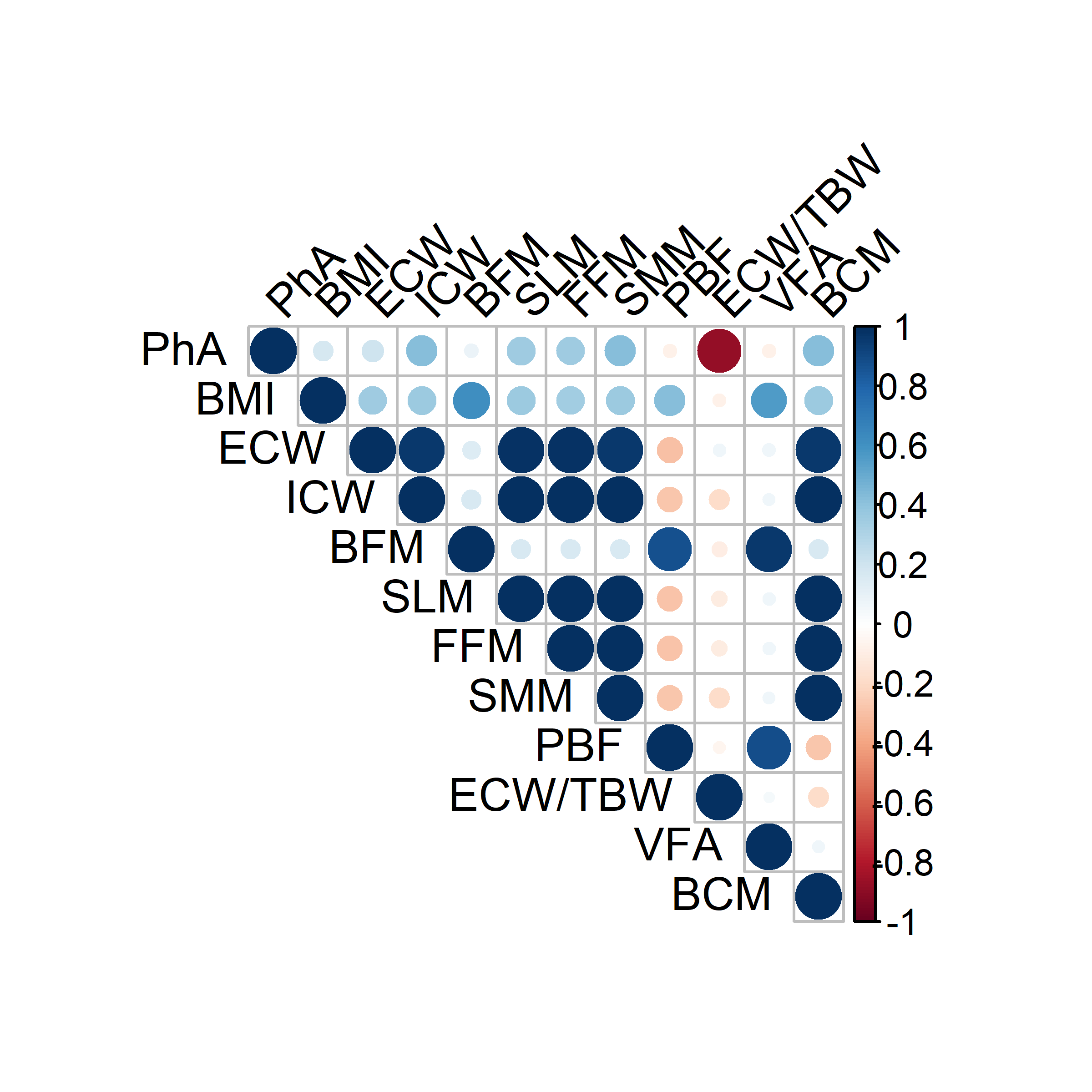


Figure legend: Heatmap of Spearman’s correlation between age, PhA and 11 other parameters obtained from body composition measurements. Colored squares represent statistically significant associations by permutation test (p < 0.01; red: positive Spearman’s rho; blue: negative Spearman’s rho).

*Abbreviations:* *PhA, phase angle; BMI, Body mass index; ECW, Extracellular Water; ICW, Intracellular Water; BFM, Body Fat Mass; SLM, Soft Lean Mass; FFM, Fat-Free Mass; SMM, Skeletal Muscle Mass; PBF, Percent Body Fat; ECW/TBW, Extracellular Water/Total body water; VFA, Visceral Fat Area; BCM, Body Cell Mass*

**Item S1 Supplementary Methods**

***Phase angle(PhA) calculation***

$$R=\sqrt{Z^{2}-Xc^{2}}$$

$$PhA=\arctan(\frac{Xc}{R})\times\frac{180^{\circ}}{\pi}$$

***Prognostic nutritional index (PNI) calculation***

$$PNI=serum\_albumi\text{n(g/L)}+5\times lymphocyte\_count(\times10^{9}/L)$$

*****Systemic inflammation response index (SIRI) calculation*****

$$SIRI=\frac{Neutrophil\_count(\times10^{9}/L)\times Monocyte\_count(\times10^{9}/L)}{Lymphocyte\_count(\times10^{9}/L)}$$

Table S1. Percentage of missing data

| Variables | Proportion of missing data | Variables | Proportion of missing data |
| --- | --- | --- | --- |
| Gender | 0.00% | BMI (kg/m^2^) | 0.00% |
| Age, years | 0.00% | SIRI | 26.07% |
| CKD stage, n(%) | 0.00% | ACR, mg/g | 29.43% |
| Hypertension, n(%) | 0.00% | PNI | 34.51% |
| Diabetes, n(%) | 0.00% | TBW (L) | 0.00% |
| Hyperlipidemia, n(%) | 0.00% | ICW (L) | 0.00% |
| Hyperuricemia, n(%) | 0.00% | ECW (L) | 0.00% |
| Cardiovascular disease, n(%) | 0.00% | BFM (kg) | 0.00% |
| Cr, μmol/L | 0.00% | SLM (kg) | 0.00% |
| eGFR, ml/min/1.73 m^2^ | 0.00% | FFM (kg) | 0.00% |
| HDL-C, mmol/L | 37.60% | SMM (kg) | 0.00% |
| LDL-C, mmol/L | 37.35% | PBF (%) | 0.00% |
| UA, mmol/L | 11.76% | ECW/TBW (%) | 0.00% |
| SBP, mmHg | 15.70% | VFA (cm^2^) | 0.00% |
| DBP, mmHg | 15.70% | BCM (kg) | 0.00% |
| MAP, mmHg | 15.70% | PhA (°) | 0.00% |

*Abbreviations: PhA, phase angle; CKD, chronic kidney disease; SBP, systolic blood pressure; DBP, diastolic blood pressure; MAP, mean arterial pressure; Cr, serum creatinine; eGFR, estimated glomerular filtration rate; LDL-C, low-density lipoprotein cholesterol; HDL-C, high-density lipoprotein cholesterol; UA, uric acid; SIRI, systemic inflammation response index; PNI, prognostic nutritional index; ACR, urine albumin/creatinine ratio; BMI, body mass index; ECW, Extracellular Water; ICW, Intracellular Water; BFM, Body Fat Mass; SLM, Soft Lean Mass; FFM, Fat-Free Mass; SMM, Skeletal Muscle Mass; PBF, Percent Body Fat; ECW/TBW, Extracellular Water/Total body water; VFA, Visceral Fat Area; BCM, Body Cell Mass*

Table S2. Variance inflation factor (VIF)

| Variables | GVIF |
| --- | --- |
| PhA | 1.71 |
| Sex | 1.37 |
| Age | 1.77 |
| Hypertension | 1.39 |
| Diabetes | 1.25 |
| Hyperlipidemia | 1.16 |
| Hyperuricemia | 1.27 |
| Cardiovascular disease | 1.09 |
| BMI | 1.11 |
| MAP | 1.06 |
| LDL | 1.18 |
| HDL | 1.25 |
| UA | 1.24 |
| PNI | 1.81 |
| SIRI | 1.11 |
| eGFR | 1.73 |
| UACR | 1.72 |

*Abbreviations: Cr, serum creatinine; eGFR, estimated glomerular filtration rate; LDL-C, low-density lipoprotein cholesterol; HDL-C, high-density lipoprotein cholesterol; UA, uric acid; BMI, body mass index; MAP, mean arterial pressure; UACR, urine albumin to creatinine ratio; SIRI, systemic inflammation response index; PNI, prognostic nutritional index .*

Table S3. Characteristics grouped by the turning point of phase angle in CKD patients

| Variables | Total | PhA ≤ 5.0 | PhA＞5.0 | *P* value |
| --- | --- | --- | --- | --- |
|  | *N=2202* | *N=1143* | *N=1059* |  |
| Man, n(%) | 1213 (55.1%) | 467 (40.9%) | 746 (70.4%) | <0.001 |
| Age, years | 52.0 (38.1-64.1) | 59.3 (45.9-68.6) | 45.6 (35.1-55.7) | <0.001 |
| CKD stage- n(%) | |  |  | <0.001 |
| 1 | 469 (21.3%) | 225 (19.7%) | 244 (23.0%) |  |
| 2 | 551 (25.0%) | 207 (18.1%) | 344 (32.5%) |  |
| 3 | 679 (30.8%) | 378 (33.1%) | 301 (28.4%) |  |
| 4 | 299 (13.6%) | 195 (17.1%) | 104 (9.82%) |  |
| 5 | 204 (9.26%) | 138 (12.1%) | 66 (6.23%) |  |
| Hypertension, n(%) | 1196 (54.3%) | 689 (60.3%) | 507 (47.9%) | <0.001 |
| Diabetes, n(%) | 469 (21.3%) | 346 (30.3%) | 123 (11.6%) | <0.001 |
| Hyperlipidemia, n(%) | 729 (33.1%) | 394 (34.5%) | 335 (31.6%) | 0.171 |
| Hyperuricemia, n(%) | 1117 (50.7%) | 545 (47.7%) | 572 (54.0%) | 0.003 |
| Cardiovascular disease, n(%) | 184 (8.36%) | 127 (11.1%) | 57 (5.38%) | <0.001 |
| BMI (kg/m^2^) | 23.0 (20.7-25.4) | 22.2 (20.1-24.6) | 23.7 (21.6-26.2) | <0.001 |
| TBW (L) | 32.7 (28.0-37.8) | 29.8 (26.5-35.1) | 35.5 (30.9-39.2) | <0.001 |
| ICW (L) | 20.0 (17.1-23.2) | 18.1 (16.1-21.1) | 22.0 (19.2-24.3) | <0.001 |
| ECW (L) | 12.7 (10.9-14.6) | 11.8 (10.4-13.9) | 13.5 (11.8-14.9) | <0.001 |
| BFM (kg) | 16.4 (12.2-21.2) | 16.0 (12.1-20.4) | 16.7 (12.5-21.9) | 0.011 |
| SLM (kg) | 41.9 (35.8-48.4) | 38.2 (33.9-44.9) | 45.6 (39.7-50.4) | <0.001 |
| FFM (kg) | 44.3 (38.0-51.2) | 40.5 (36.1-47.5) | 48.3 (42.0-53.2) | <0.001 |
| SMM (kg) | 24.1 (20.2-28.2) | 21.7 (19.0-25.5) | 26.7 (23.0-29.7) | <0.001 |
| PBF (%) | 27.1±8.43 | 27.9±8.67 | 26.2±8.07 | <0.001 |
| ECW/TBW (%) | 0.39 (0.38-0.39) | 0.39 (0.39-0.40) | 0.38 (0.38-0.38) | <0.001 |
| VFA (cm^2^) | 72.3 (53.7-98.9) | 73.9 (56.1-101) | 71.2 (51.5-96.6) | 0.002 |
| BCM (kg) | 28.7 (24.4-33.2) | 26.0 (23.1-30.2) | 31.5 (27.4-34.8) | <0.001 |
| PhA(°) | 5.00 (4.50-5.60) | 4.50 (4.10-4.80) | 5.60 (5.30-6.00) | 0.000 |
| SBP, mmHg | 127 (120-136) | 130 (120-140) | 125 (118-133) | <0.001 |
| DBP, mmHg | 75.0 (69.0-80.0) | 75.0 (69.0-80.0) | 76.0 (70.0-80.1) | 0.012 |
| MAP, mmHg | 92.7 (86.7-98.3) | 93.0 (86.7-99.3) | 92.4 (86.3-97.9) | 0.072 |
| Cr, μmol/L | 114 (83.0-176) | 118 (80.0-202) | 111 (86.0-150) | 0.049 |
| eGFR, ml/min/1.73 m^2^ | 55.4 (31.6-84.2) | 48.5 (24.9-79.9) | 64.0 (42.1-86.7) | <0.001 |
| HDL-C, mmol/L | 1.32 (1.14-1.51) | 1.34 (1.17-1.55) | 1.30 (1.12-1.48) | <0.001 |
| LDL-C, mmol/L | 3.30 (2.84-3.80) | 3.33 (2.84-3.85) | 3.28 (2.83-3.77) | 0.117 |
| UA, mmol/L | 421 (357-481) | 416 (348-473) | 424 (363-489) | 0.013 |
| Alb, g/L | 43.1 (39.0-45.8) | 41.4 (36.3-44.5) | 44.6 (41.2-46.9) | <0.001 |
| PNI | 52.8 (48.2-56.6) | 51.0 (45.0-54.8) | 54.1 (51.0-58.0) | <0.001 |
| SIRI | 1.00 (0.651.55) | 1.04 (0.65-1.62) | 0.96 (0.651.47) | 0.082 |
| UACR, mg/g | 715 (181-1935) | 1071 (292-2660) | 468 (143-1186) | <0.001 |

The values for categorical variables are given as numbers (percentage); values for continuous variables are given as median [interquartile range] or mean ± standard deviation.

*Abbreviations: PhA, phase angle; CKD, chronic kidney disease; SBP, systolic blood pressure; DBP, diastolic blood pressure; MAP, mean arterial pressure; Cr, serum creatinine; eGFR, estimated glomerular filtration rate; LDL-C, low-density lipoprotein cholesterol; HDL-C, high-density lipoprotein cholesterol; UA, uric acid; SIRI, systemic inflammation response index; PNI, prognostic nutritional index; UACR, urine albumin/creatinine ratio;Alb, serum albumin; BMI, body mass index; ECW, Extracellular Water; ICW, Intracellular Water; BFM, Body Fat Mass; SLM, Soft Lean Mass; FFM, Fat-Free Mass; SMM, Skeletal Muscle Mass; PBF, Percent Body Fat; ECW/TBW, Extracellular Water/Total body water; VFA, Visceral Fat Area; BCM, Body Cell Mass*

Table S4. Characteristics grouped by phase angle trajectories of CKD patients

| Variables | Total | 1 | 2 | 3 | 4 | *P* value |
| --- | --- | --- | --- | --- | --- | --- |
|  | *N=1114* | *N=196* | *N=491* | *N=341* | *N=86* |  |
| Man | 577(51.8%) | 75(38.3%) | 187(38.1%) | 234(68.6%) | 81(94.2%) | <0.001 |
| Survival time, days | 1126(703-1598) | 977(552-1525) | 1120(704-1620) | 1155(773-1624) | 1268(801-1534) | 0.068 |
| Age, years | 51.1(37.5-62.8) | 62.2(52.9-71.9) | 54.3(41.5-64.6) | 43.1(33.8-54.0) | 38.7(31.9-45.9) | <0.001 |
| CKD stage, n(%) | |  |  |  |  | <0.001 |
| 1 | 254(22.8%) | 37(18.9%) | 107(21.8%) | 93(27.3%) | 17(19.8%) |  |
| 2 | 278(25.0%) | 29(14.8%) | 108(22.0%) | 99(29.0%) | 42(48.8%) |  |
| 3 | 373(33.5%) | 85(43.4%) | 161(32.8%) | 107(31.4%) | 20(23.3%) |  |
| 4 | 141(12.7%) | 30(15.3%) | 76(15.5%) | 32(9.38%) | 3(3.49%) |  |
| 5 | 68(6.10%) | 15(7.65%) | 39(7.94%) | 10(2.93%) | 4(4.65%) |  |
| Hypertension, n(%) | 626(56.2%) | 137(69.9%) | 277(56.4%) | 172(50.4%) | 40(46.5%) | <0.001 |
| Diabetes, n(%) | 200(18.0%) | 66(33.7%) | 91(18.5%) | 33(9.68%) | 10(11.6%) | <0.001 |
| Hyperlipidemia, n(%) | 456(40.9%) | 100(51.0%) | 194(39.5%) | 131(38.4%) | 31(36.0%) | 0.015 |
| Hyperuricemia, n(%) | 638(57.3%) | 122(62.2%) | 260(53.0%) | 201(58.9%) | 55(64.0%) | 0.053 |
| Cardiovasculardisease, n(%) | 90(8.08%) | 30(15.3%) | 40(8.15%) | 15(4.40%) | 5(5.81%) | <0.001 |
| Cr, μmol/L | 111(81.0-162) | 116(86.8-173) | 110(76.0-174) | 110(81.0-147) | 113(98.0-129) | 0.595 |
| eGFR, ml/min/1.73m^2^ | 57.8(36.3-86.1) | 47.5(30.7-72.3) | 53.4(32.0-84.9) | 64.0(43.8-95.7) | 69.4(56.7-79.3) | <0.001 |
| HDL-C, mmol/L | 1.33(1.12-1.54) | 1.41(1.15-1.66) | 1.34(1.17-1.54) | 1.29(1.10-1.49) | 1.25(1.07-1.41) | <0.001 |
| LDL-C, mmol/L | 3.28(2.75-3.80) | 3.33(2.68-3.91) | 3.21(2.72-3.80) | 3.33(2.78-3.80) | 3.21(2.83-3.63) | 0.463 |
| UA, mmol/L | 414(352-476) | 414(343-475) | 407(344-467) | 420(363-482) | 417(358-496) | 0.106 |
| Alb, g/L | 43.6 (40.1-46.2) | 40.9 (36.9-44.3) | 43.4 (39.7-45.4) | 44.9 (41.8-47.4) | 46.1 (42.1-48.1 | <0.001 |
| PNI | 53.3 (49.4-57.0) | 50.1 (45.1-54.6) | 52.7 (49.1-55.9) | 55.0 (52.2-58.4) | 56.6 (52.6-60.1) | <0.001 |
| SIRI | 0.96 (0.66-1.47) | 0.98 (0.65-1.59) | 0.91 (0.63-1.39) | 1.01 (0.68-1.53) | 1.04 (0.74-1.45) | 0.127 |
| UACR, mg/g | 602 (173-1495) | 1129 (200-2907) | 758 (213-1532) | 462 (130-1054) | 381 (154-733) | <0.001 |
| BMI (kg/m^2^) | 22.7(20.7-25.2) | 21.9(20.1-24.1) | 22.3(20.4-24.7) | 23.8(21.4-26.1) | 24.3(22.4-26.2) | <0.001 |
| TBW(L) | 32.3(27.7-37.3) | 28.7(25.9-35.0) | 30.2(26.8-34.4) | 35.3(30.9-38.7) | 38.0(35.7-41.2) | <0.001 |
| ICW(L) | 19.8(17.0-22.9) | 17.3(15.6-20.9) | 18.4(16.4-21.2) | 21.8(19.1-23.9) | 23.8(22.3-25.7) | <0.001 |
| ECW(L) | 12.4(10.7-14.3) | 11.4(10.1-14.0) | 11.7(10.4-13.4) | 13.4(11.9-14.8) | 14.2(13.3-15.5) | <0.001 |
| BFM (kg) | 16.4(12.5-20.9) | 16.0(12.5-20.9) | 16.2(12.6-20.4) | 16.8(12.0-21.9) | 16.2(12.5-21.3) | 0.884 |
| SLM (kg) | 41.3(35.5-47.8) | 36.6(33.0-44.5) | 38.6(34.3-44.1) | 45.2(39.6-49.7) | 48.9(46.0-53.0) | <0.001 |
| FFM (kg) | 43.9(37.6-50.6) | 38.9(35.1-47.3) | 41.0(36.4-46.7) | 47.9(42.0-52.6) | 51.8(48.6-56.1) | <0.001 |
| SMM (kg) | 23.8(20.1-27.9) | 20.5(18.3-25.2) | 22.0(19.4-25.6) | 26.4(22.9-29.2) | 29.0(27.1-31.4) | <0.001 |
| PBF (%) | 27.4(8.09) | 28.9(8.95) | 28.3(7.68) | 26.1(8.13) | 24.0(6.30) | <0.001 |
| ECW/TBW (%) | 0.39(0.38-0.39) | 0.40(0.39-0.40) | 0.39(0.38-0.39) | 0.38(0.38-0.38) | 0.38(0.37-0.38) | <0.001 |
| VFA (cm^2^) | 72.3(54.2-97.0) | 75.5(60.4-108) | 72.0(55.1-96.5) | 71.9(48.9-96.7) | 67.8(47.0-90.8) | 0.010 |
| BCM (kg) | 28.4(24.3-32.8) | 24.8(22.3-29.9) | 26.4(23.5-30.3) | 31.2(27.4-34.3) | 34.0(32.0-36.7) | <0.001 |
| PhA (°) | 5.10(4.60-5.60) | 4.10(3.80-4.40) | 4.90(4.70-5.10) | 5.70(5.40-5.90) | 6.30(6.02-6.60) | <0.001 |
| SBP, mmHg | 127(120-135) | 130(122-139) | 126(119-136) | 126(118-132) | 126(119-131) | <0.001 |
| DBP, mmHg | 75.5(70.0-80.0) | 75.0(70.0-81.0) | 74.5(69.0-80.0) | 77.5(70.0-81.0) | 78.0(71.0-80.7) | 0.001 |
| MAP, mmHg | 92.7(86.7-97.9) | 93.6(88.2-99.7) | 91.7(86.0-97.0) | 93.3(86.7-98.0) | 93.3(89.3-97.3) | 0.012 |

The values for categorical variables are given as numbers (percentage); values for continuous variables are given as median [interquartile range] or mean ± standard deviation.

*Abbreviations: PhA, phase angle; CKD, chronic kidney disease; SBP, systolic blood pressure; DBP, diastolic blood pressure; MAP, mean arterial pressure; Cr, serum creatinine; eGFR, estimated glomerular filtration rate; LDL-C, low-density lipoprotein cholesterol; HDL-C, high-density lipoprotein cholesterol; UA, uric acid; SIRI, systemic inflammation response index; PNI, prognostic nutritional index; UACR, urine albumin/creatinine ratio; Alb, serum albumin; BMI, body mass index; ECW, Extracellular Water; ICW, Intracellular Water; BFM, Body Fat Mass; SLM, Soft Lean Mass; FFM, Fat-Free Mass; SMM, Skeletal Muscle Mass; PBF, Percent Body Fat; ECW/TBW, Extracellular Water/Total body water; VFA, Visceral Fat Area; BCM, Body Cell Mass*

Table S5. The final four-group trajectory model of phase angle level as function of observation time in CKD patients

1. Estimation of Group Trajectory Model Parameters

| **Group** | ***Avepp(%)*** | ***OCC*** | ***P_j_(%)*** | ***π_j_(%)*** | ***BIC^#2^*** | ***△BIC^#2^*** | ***E_k_*** |
| --- | --- | --- | --- | --- | --- | --- | --- |
|  |  |  |  |  | -3726.49 | 380.60 | 0.885 |
| ***Group1*** | 94.826 | 83.5 | 17.594 | 18.000 |  |  |  |
| ***Group 2*** | 93.189 | 17.7 | 44.075 | 43.559 |  |  |  |
| ***Group 3*** | 92.367 | 27.6 | 30.610 | 30.449 |  |  |  |
| ***Group 4*** | 93.657 | 170.0 | 7.720 | 7.992 |  |  |  |

Good model fit is indicated by: (1) Odds of correct classification (OCC) greater than 5 for each class, and (2) A good consistency between the posterior probabilities (Pj) of group membership and the population distribution proportion of group membership (πj).

*Abbreviations: Avep%, Average posterior probability; Pj, Posterior probability; πj, Population Distribution Proportion; BIC^#2^, Bayesian Information Criterion; ∆BIC^#2^, BIC difference between the two models; Ek, Relative entropy (Ek)*

1. Evaluation of the Fitting Effect of Each Trajectory Group within the Group Trajectory Model

| **Group** | **Parameter** | ***β*** | ***SE*** | ***t*** | ***P*** |
| --- | --- | --- | --- | --- | --- |
| Group 1 | Intercept | 4.08747 | 0.03054 | 133.840 | 0.00000 |
|  | Linear | -0.01202 | 0.00598 | -2.010 | 0.04448 |
| Group 2 | Intercept | 4.90022 | 0.02125 | 230.599 | 0.00000 |
|  | Linear | -0.01511 | 0.00356 | -4.244 | 0.00002 |
| Group 3 | Intercept | 5.61649 | 0.01829 | 307.080 | 0.00000 |
| Group 4 | Intercept | 6.19952 | 0.06017 | 103.033 | 0.00000 |
|  | Linear | 0.09581 | 0.02884 | 3.322 | 0.00090 |
|  | Quadratic | -0.00606 | 0.00293 | -2.068 | 0.03866 |

Note: significant level was set at *P*≤0.05.

Table S6 Variation in the mean phase angle in different stages of CKD

| CKD stage | Phase angle (mean±sd) | *P* | *P for trend* | *P (3b vs 4)* | *P (3b vs 5)* | *P(4 vs 5)* |
| --- | --- | --- | --- | --- | --- | --- |
| 1-3a | 5.17±0.79 | <0.001 | / |  |  |  |
| 3b-5 | 4.79±0.82 |  |  |  |  |  |
| 1 | 5.07±0.77 | <0.001 | <0.001 | 0.382 | 0.022 | 0.209 |
| 2 | 5.32±0.79 |  |  |  |  |  |
| 3a | 5.13±0.77 |  |  |  |  |  |
| 3b | 4.87±0.78 |  |  |  |  |  |
| 4 | 4.79±0.82 |  |  |  |  |  |
| 5 | 4.67±0.84 |  |  |  |  |  |

*Abbreviations: CKD, chronic kidney disease*

Table S7 Distributions of the composite endpoint

For 570 participants reaching composite endpoint, proportion and types of clinical outcomes (strictly limited to the first event per participant) are as follows:

- ＞30% decline of eGFR: 258 cases (45.3%)
- ＜5ml/min/1.73m² in eGFR: 160 cases (28.1%)
- Doubled of SCr from the baseline: 121 cases (21.2%)
- Renal replacement therapy: 17 cases (hemodialysis [n=8], peritoneal dialysis [n=7], kidney transplantation [n=2]) (3.0%)
- All-cause mortality: 14 cases (2.4%)
